# Gender Evaluation and Numeric Distribution in Emergency Medicine Residencies (GENDER): A Retrospective Analysis of Gender Ratios Among Residents and Residency Directors from 2014–2017

**DOI:** 10.5811/westjem.2022.7.54678

**Published:** 2022-10-24

**Authors:** Ryan Gibney, Christina Cantwell, Shannon Toohey, Megan Boysen-Osborn, Warren Wiechmann, Soheil Saadat, Angela Allen, Alisa Wray

**Affiliations:** * University of California, Irvine, Department of Emergency Medicine, Orange, California; † University of North Carolina, Department of Emergency Medicine, Chapel Hill, North Carolina

## Abstract

**Introduction:**

While females make up more than half of medical school matriculants, they only comprise about one third of emergency medicine (EM) residents. We examined EM residency cohorts with entering years of 2014–2017 to estimate the ratio of males to females among residents and program leadership to determine what correlation existed, if any, between program leadership and residency gender distributions.

**Methods:**

We identified 171 accredited EM residency programs in the United States with resident cohorts entering between 2014–2017 with publicly available data that were included in the study. The number of male and female residents and program directors were counted. We then confirmed the counts by contacting the programs directly to confirm accuracy of the data collected from program websites.

**Results:**

Within the included 171 programs, the overall male to female EM resident ratio was 1.78:1. Individual program ratios ranged from 0.85–8.0. Only eight programs (5.6%) had a female-predominant ratio. Among program directors, the overall male to female ratio was 2.17:1. TThe gender of the program director did not have a statistically significant correlation with the male to female ratio among its residents (P = .93).

**Conclusion:**

Within 171 residency programs across the US with entering cohorts between 2014–2017, the average male to female ratio among residents is nearly 2:1. No significant correlation exists between the gender distribution among a program’s leadership and its residents.

## INTRODUCTION

While females make up more than half of medical school matriculants, they comprise only about one third of emergency medicine (EM) residents^1^; this percentage has remained relatively stagnant over the past 10 years.^2^ Men comprise a higher percentage of practicing emergency physicians,^3^ and it has been shown that within EM, male and female residents demonstrate implicit bias favoring male leadership.^4^ While several factors influence students’ decisions in applying to residency programs, the influence of gender composition within a given program has not been well studied, with limited literature on the topic. In this study we examined publicly available data to determine the baseline gender makeup of EM residencies as a proxy for the specialty. We then compared the gender distribution of EM residents and program leadership from entering years 2014–2017 to determine whether there was a relationship between the composition of a residency class and its leadership.

## METHODS

We identified 171 Accreditation Council for Graduate Medical Education (ACGME)-accredited EM residency programs in the United States in 2017.^5^ We chose residency cohorts with entering years 2014–2017 for inclusion. We selected cohort data by entering year rather than graduating year to account for 3- and 4-year programs and to accurately capture the true variance of gender year by year. The institutional review board determined that the study was exempt for public data collection and did not require informed consent.

We used publicly available data from individual program websites to determine resident gender distribution. We manually counted the gender distribution of residents at each program with publicly available pictures of the program’s residents and faculty on their websites. We contacted programs without publicly available data via email to determine their gender distribution. Once the data was collected, we contacted program directors and coordinators via email to verify the accuracy of the manually collected data. We allowed 60 days for response, with follow-up emails to those with no response.

A total of 47 programs verified the data, which were entered into a matrix-style data collection instrument for analysis. By using this approach, we found that most of the data collected from publicly available program websites was accurate; so, we chose to also include both verified and unverified data in our analysis. Public data was determined to be relatively accurate with a 1.65% error on the total number of residents reported, a 2.08% error on the number of male residents reported, and a 3.73% error on the number of female residents reported. We calculated the percent error using datasets from programs that confirmed our manually collected data. The calculation was performed by subtracting the estimated (manually counted) number of total residents or male residents only or female residents only from the actual (confirmed) data for these categories, and then dividing by the actual data. Percent errors were then averaged for each category.

We identified program directors, and associate and assistant program directors using similar methods as above. We confirmed gender distribution among those identified using email, and the gender makeup of program leadership was confirmed with the program coordinator. A total of 47 programs verified their residency leadership data.

We analyzed the data using simple ratios to determine the overall gender distribution within all identified EM programs. The ratios for individual residency cohort years 2014–2017 were compared against one another in addition to comparison against the cumulative ratio for the four-year period. Additionally, we analyzed individual programs to determine the variation from the mean. The data from individual programs was compared to that of the program leadership to determine whether there was a correlation between the gender of the leadership and the overall makeup of the residency cohorts with respect to the mean.

## RESULTS

Public data were available for 171 ACGME-accredited EM residency programs in the US. Of the 7,185 residents identified, 4,598 (64%) were male compared to 2,587 (36%) female, giving an overall male to female (M:F) ratio of 1.78:1. This is similar to 2017 Association of American Medical Colleges (AAMC) data for all EM residents (35.5% female, M:F 1.81:1). We examined individual programs and found gender ratios among residents to range from 0.85–8.0 (Table). Of the 171 programs examined, only eight (5.6%) had a female-predominant dichotomy.

Gender distribution among program directors was similar to that of residents, showing 117 males and 54 females (2.17:1 ratio) and slightly less dichotomous than a previous report.^6^ When evaluated based on the gender of the program director, male to female resident ratio was 2.11 (SD = 0.91; 95% confidence interval [CI] 1.65–2.56) for programs with a female program director and 2.07 (SD = 0.81; 95% CI 1.87–2.28) for programs with a male program director. Thus, there was no statistically significant difference in the resident gender ratio based on the gender of the program director when comparing the two with a Mann-Whitney U Test (P = .93).

## DISCUSSION

Throughout this paper, we use the term “gender” to refer to male or female. We recognize and respect that gender exists on a non-binary spectrum. Since the first stage of our data collection was created from publicly available information on program websites, we focused on determining only the male:female resident and program leadership ratios across the residency programs that existed in 2017. We recognize this binary categorization as a limitation of our study. Additionally, the AAMC reports the breakdown of residents by gender as “male” or “female,” categorized in a binary manner.^1^,^3^ While we recognize this dichotomy as a limited view, this binary categorization is also how gender is defined within the sphere of residency programs in the AAMC resident report.

Other residency resources such as the American Medical Association’s FREIDA database for residency programs by specialty also categorize programs’ current resident distribution as male or female.^7^ While we recognize that more consistent and proper use of the terms “gender” and “sex” are needed across reporting platforms, the binary categorization is all that was available from public data. Standardized reporting of gender as describing one’s own identity vs the term sex to describe “male” or “female” is necessary on a systemic level to more accurately capture the true composition of residency programs, and further research can be conducted when such data is available. As a next step, a follow-up survey can be sent directly to current residents to capture the true gender diversity in EM residencies by having an interface in which respondents can self-identify.

The AAMC releases biennial data on gender distribution; however, this study provides further information on how the gender distribution varies across EM residencies. While the overall AAMC reported breakdown of gender in EM was 1.82:1 at the time of this data collection,^1^ our results were consistent with this with an overall ratio of 1.78:1. However, the wide variance with some programs as much as 8.0:1 M:F is not clearly evident in generalized data such as this and may have wide-reaching implications on residency selection and training.

DeFazio published similar findings in 2017 regarding gender distribution of residents and program director.^6^ Our study differs in that we examined resident classes with entering years 2014–2017 to account for three- and four-year programs vs the graduating classes as in DeFazio’s work. We found the percentage of female residents to be approximately 36%, in congruence with DeFazio’s estimation of 40%. Additionally, we compared the ratio trends within programs, finding that resident gender ratios did not differ significantly depending on program leadership composition. Our findings on gender diversity within EM residency leadership were consistent with previous data showing 76% of programs with male directors^6^; however, direct influence of program director and faculty gender had not previously been evaluated.

## LIMITATIONS

The use of publicly available data as a proxy to estimate the ratio of males to females in EM residencies is limited by only partial confirmation of the identified programs. Additionally, there were some instances in which a program’s director changed from 2014–2017, which altered the gender ratio of program leadership.

## CONCLUSION

While approximately half of medical school graduates are female, females comprise only about one third of EM residents. Current data shows a clear gender discrepancy within the specialty of EM, both in resident and leadership populations. This study identified the current discrepancies in EM residencies across the country showing an overall ratio of 1.78:1. We identified only eight programs with a female-predominant ratio of residents out of a cohort of 171 EM residency programs. Among program leadership, the number of males again predominated with a slightly higher than two-thirds to one-third ratio. The distribution of male and female residents does not appear to differ significantly when compared to the gender distribution of program leadership. Further studies are warranted to determine which program characteristics influence medical students’ decisions in choosing a residency program and whether existing gender distributions play a role in an applicant’s decision-making process.

**Population Health Research Capsule**

What do we already know about this issue?

*While females make up more than half of medical school matriculants, they comprise only about one third of emergency medicine (EM) residents*.

What was the research question?

*We sought to determine what correlation existed, if any, between program leadership and residency gender distributions*.

What was the major finding of the study?

*The average resident male:female ratio is nearly 2:1. No significant correlation exists between the gender distribution of a program’s leadership and its residents*.

How does this improve population health?

*Understanding gender make-up of programs provides a baseline for future studies, to determine whether existing gender distributions play a role in an applicant’s decision-making process*.

## Figures and Tables

**Table:** Frequency of gender ratios among emergency medicine (EM) residents from 2014–2017 from 171 EM programs. Ratios listed as male:female (M:F).

Ratio (M:F)	Frequency	Percent
< 1.00	8	4.7%
1.00	4	2.3%
1.01–2.00	87	50.9%
2.01–3.00	46	26.9%
> 3.00	26	15.2%
Total	171	100%
